# Deceased donor neutrophil gelatinase-associated lipocalin and delayed graft function after kidney transplantation: a prospective study

**DOI:** 10.1186/cc10220

**Published:** 2011-05-05

**Authors:** Maria E Hollmen, Lauri E Kyllönen, Kaija A Inkinen, Martti LT Lalla, Jussi Merenmies, Kaija T Salmela

**Affiliations:** 1 Division of Transplantation, Helsinki University Hospital, Kasarmikatu 11, 00130 Helsinki, Finland; 2 HUSLAB, Helsinki University Hospital, Surgical Hospital, Kasarmikatu 11, 00130, Helsinki, Finland; 3 Clinical Laboratory, Finnish Red Cross Blood Service, Kivihaantie 7, 00310, Helsinki, Finland

## Abstract

**Introduction:**

Expanding the criteria for deceased organ donors increases the risk of delayed graft function (DGF) and complicates kidney transplant outcome. We studied whether donor neutrophil gelatinase-associated lipocalin (NGAL), a novel biomarker for acute kidney injury, could predict DGF after transplantation.

**Methods:**

We included 99 consecutive, deceased donors and their 176 kidney recipients. For NGAL detection, donor serum and urine samples were collected before the donor operation. The samples were analyzed using a commercial enzyme-linked immunosorbent assay kit (serum) and the ARCHITECT method (urine).

**Results:**

Mean donor serum NGAL (S-NGAL) concentration was 218 ng/mL (range 27 to 658, standard deviation (SD) 145.1) and mean donor urine NGAL (U-NGAL) concentration was 18 ng/mL (range 0 to 177, SD 27.1). Donor S-NGAL and U-NGAL concentrations correlated directly with donor plasma creatinine levels and indirectly with estimated glomerular filtration rate (eGFR) calculated using the modification of diet in renal disease equation for glomerular filtration rate. In transplantations with high (greater than the mean) donor U-NGAL concentrations, prolonged DGF lasting longer than 14 days occurred more often than in transplantations with low (less than the mean) U-NGAL concentration (23% vs. 11%, *P *= 0.028), and 1-year graft survival was worse (90.3% vs. 97.4%, *P *= 0.048). High U-NGAL concentration was also associated with significantly more histological changes in the donor kidney biopsies than the low U-NGAL concentration. In a multivariate analysis, U-NGAL, expanded criteria donor status and eGFR emerged as independent risk factors for prolonged DGF. U-NGAL concentration failed to predict DGF on the basis of receiver operating characteristic curve analysis.

**Conclusions:**

This first report on S-NGAL and U-NGAL levels in deceased donors shows that donor U-NGAL, but not donor S-NGAL, measurements give added value when evaluating the suitability of a potential deceased kidney donor.

## Introduction

Deceased kidney donors are expected to have healthy kidneys which will function well in the recipient after transplantation. However, a considerable number of kidney transplantations from deceased donors are complicated by delayed graft function (DGF). There is no consensus on the ultimate effect of short DGF, lasting less than one week, on graft survival; however, when the duration of allograft dysfunction becomes prolonged, the negative effect on kidney graft survival becomes evident [1,2]. The criteria for deceased donors have been expanded because of organ shortages, and consequently DGF has become more common [3,4]. At our center, we have expanded our criteria for acceptable kidney donors since 1995. During the past ten years, the rate of DGF in transplantations from expanded criteria donors (ECDs) has been 42%, compared to 23% in transplantations from standard criteria donors (*P *= 0.001; unpublished data, Helsinki University Hospital, Division of Transplantation, Kyllönen L and Salmela K).

The quality of donor kidneys has a clear impact on long-term kidney allograft outcomes [5-7]. Various algorithms have been designed for the evaluation of deceased donors [8-10]. As these scoring systems also use recipient and transplantation variables such as cold ischemia time and human leukocyte antigen (HLA) matching, they cannot be used when deciding whether to accept or reject the donor. In practice, the judgment relies on the only readily available markers: diuresis and plasma creatinine level.

Neutrophil gelatinase-associated lipocalin (NGAL) is a new marker for acute kidney injury (AKI) which has been studied after cardiac surgery, liver transplantation and contrast media administration, as well as in intensive care unit (ICU) patients (in heterogeneous patient groups and in patients with septic vs. nonseptic AKI), in unselected patients who present to the emergency department and in critically ill multiple trauma patients [11-22]. So far, very little is known about NGAL after kidney transplantation [23-26], and there are no published data available on NGAL in deceased kidney donors. We recently found that recipient urine NGAL (U-NGAL) measured the first morning following transplantation predicted DGF, particularly in cases where early graft function (EGF) was expected on the basis of diuresis and decreasing plasma creatinine concentration [27]. In addition, recipient U-NGAL could predict DGF lasting longer than two weeks [27].

Plasma creatinine level is known to be a poor early detector of AKI. Thus, a simple laboratory test revealing AKI early on would be useful for clinicians taking care of potential donors in ICU when evaluating the quality of their kidneys. In this prospective study, we wanted to examine (1) the levels of serum NGAL (S-NGAL) and U-NGAL in deceased kidney donors, (2) whether donor S-NGAL and/or U-NGAL could be used as predictors of DGF and especially (3) prolonged DGF after kidney transplantation.

## Materials and methods

### Study design and patients

The present study was performed at Helsinki University Hospital, which provides organ transplant service for Finland, which has a population of 5.2 million. For this study, we prospectively enrolled 99 consecutive, deceased, heartbeating donors and their 176 adult kidney recipients between August 2007 and December 2008. The study protocol was approved by the Helsinki University Hospital Ethics Committee and the hospital's Department of Surgery. Written informed consent was obtained from the recipients before enrollment.

Altogether 198 kidneys were obtained from the 99 donors. One kidney was not transplanted because of a vascular lesion. Twenty-one kidneys were not included in the study: six were used for pediatric recipients, two were used for recipients who underwent combined kidney and liver transplantation and one was used for a combined kidney and lung transplantation. Nine kidneys were shipped to the other Nordic countries according to the Scandiatransplant exchange rules. Three patients did not consent to participate in the study. The recipients of the remaining 176 kidneys were included in this study.

Donor clinical history data were obtained from the hospital records. The following variables were gathered: age, gender, history of hypertension, need for cardiopulmonary resuscitation, need for intracranial surgery, use of vasopressor support, use of antidiuretic hormone (ADH), plasma creatinine level, length of hospital stay before brain death diagnosis, cause of death and multiorgan or kidney-only donation. Estimated glomerular filtration rate (eGFR) was calculated using the modification of diet in renal disease equation for glomerular filtration rate (MDRD equation) [28] in 96 adult donors. In three donors who were under 18 years of age (ages 9, 16 and 17 years), eGFR was calculated using the Schwartz formula [29]. ECDs were defined according to the criteria described by Port *et al. *[7], which include all donors older than 60 years of age, or donors older than 50 years of age with at least two of the following: plasma creatinine concentration above 132 μmol/L (1.5 mg/dL), cerebrovascular accident as the cause of death or a history of hypertension.

Intravenous steroids were given to all donors before undergoing the organ retrieval operation, and they were given mannitol before *in situ *perfusion was initiated. The University of Wisconsin solution was used for *in situ *perfusion and cold storage preservation of the kidneys. A biopsy for histological evaluation was taken from the donor kidney before the initiation of *in situ *perfusion. The biopsies were examined later and scored using the Banff 97 criteria [30] and the Chronic Allograft Damage Index (CADI) [31] to quantify renal allograft histology. In both scoring systems, different components in the biopsy are semiquantitatively evaluated and then summarized. The CADI score may have a value between 0 and 18, and it is obtained from individual component scores (0 to 3) for glomerular sclerosis, vascular intimal proliferation, interstitial inflammation, mesangial matrix increase, tubular atrophy and interstitial fibrosis.

Recipient clinical data were obtained from the patients' hospital records and the Finnish Kidney Transplant Registry database. Plasma creatinine concentration was recorded daily after transplantation during the recipient's stay in the transplant unit, then at 3 months and 1 year after transplantation. eGFR was calculated using the MDRD equation [28] at 3 months and 1 year after transplantation. Our standard immunosuppressive regimen was used as previously described [27].

The primary recipient outcome variable was onset of graft function after transplantation. DGF was defined as described by Halloran *et al. *[32]: oliguria less than 1 L/24 hours for more than 2 days, or plasma creatinine concentration greater than 500 μmol/L throughout the first week after transplantation, or more than one dialysis session needed during the first week after transplantation. In the analyses examining DGF duration, we divided the transplantations into three groups: EGF (*n *= 106), short DGF lasting less than 14 days (*n *= 43) and prolonged DGF lasting 14 days or longer (*n *= 27).

### NGAL sample collection and detection

Serum samples for NGAL analyses were taken for logistical reasons in the donor hospital simultaneously with blood samples for HLA determination. The serum sample was drawn before the diagnosis of brain death in 36 cases (mean 9.1 hours, range 0.5 to 24.5) and after that in 63 cases (mean 1.4 hours, range 0.03 to 5.6). The serum samples were taken before steroid administration in 77 donors and after that in 22 donors. Urine samples were taken by the transplant team at the beginning of donor surgery. Thus all donors had already received the steroids before their urine samples were taken. All samples were immediately centrifuged at 2,500 rpm at 4°C for 10 minutes, and after that the serum and urine supernatant were divided into tubes and frozen at -70°C. No additives were used.

The S-NGAL assays were performed using a commercial enzyme-linked immunosorbent assay (ELISA) kit (BioPorto Diagnostics A/S, Gentofte, Denmark) as recommended by the manufacturer. The measurements were performed in duplicate and blinded to sample sources and clinical outcomes. Serum samples were available for NGAL analyses from 95 donors. In four cases, S-NGAL levels could not be analyzed because of inadequate (*n *= 2) or incorrectly processed (*n *= 2) sampling.

The U-NGAL assays were performed using a standardized clinical platform (ARCHITECT analyzer; Abbott Diagnostics, Abbott Park, IL, USA) as previously described [33]. Urine samples from 95 donors were available for NGAL analyses. Donor U-NGAL levels could not be determined in four cases because of inadequate (*n *= 1) or incorrectly processed (*n *= 3) sampling.

We divided the donors using the mean NGAL concentrations as cutoffs into a high NGAL group (S-NGAL ≥214 ng/mL, *n *= 38; U-NGAL ≥18 ng/mL, *n *= 26) and a low NGAL group (S-NGAL < 214 ng/mL, *n *= 57; U-NGAL < 18 ng/mL, *n *= 69).

### Statistical analyses

SPSS version 18.0 software (SPSS, Inc., Chicago, IL, USA) was used for statistical analyses. All analyzed variables were tested for distribution. Student's *t*-test and analysis of variance were used to calculate samples with normal distribution, and the Mann-Whitney *U *and Kruskal-Wallis tests were used for analyses of samples with skewed distribution. χ^2 ^and Fisher's exact tests were employed for analyses of contingency tables. To assess DGF predictors, multilogistic regression analyses (forward and conditional) were used. Factors which were significantly different between the DGF and EGF groups in the univariate analyses, as well as for the other clinically relevant factors in this respect, were included in the multivariate analyses. The factors in the multivariate analyses consisted of categorical variables and the covariates of continuous variables. The parametric correlations were assessed using the Pearson correlation coefficient, and the nonparametric correlations were assessed using the Spearman correlation coefficient. Receiver operating characteristic curve (ROC) analysis was performed to assess the potential of NGAL to predict DGF. Positive and negative predictive values were calculated using Bayes' formula. A *P *value < 0.05 was considered significant.

## Results

Table 1 shows the donor characteristics, and Table 2 shows the recipient characteristics and transplantation details. After transplantation, DGF occurred in 70 (39.8%) of 176 cases. The mean time to onset of graft function in the DGF transplantations was 12.0 days after transplantation (range 3 to 38 days, SD 7.0). Of the 70 DGF transplantations, 26 (37.1%) had prolonged DGF lasting 14 days or longer. Graft survival at 1 year was 99.1% in the EGF group, 100% in the short DGF group and 73.1% in the prolonged DGF group (*P *= 0.001). Acute rejection occurred in 10 (5.7%) of 176 transplantations at a mean of 16.8 days after transplantation (range 7 to 49 days, SD 12.6).

**Table 1:** Clinical characteristics of 99 deceased kidney donors^a^

Clinical characteristics	Statistics
Mean age, years (± SD)	51.8 (± 13.7)
Gender, *n *(%)	
Female	43 (43.4%)
Male	56 (56.6%)
Cause of death, *n *(%)	
Cerebrovascular accident	74 (74.7%)
Traumatic brain injury	25 (25.3%)
Mean plasma creatinine, μmol/L (± SD)	62 (± 19.4)
Mean eGFR, mL/min (± SD)	116 (± 34.8)
History of hypertension, *n *(%)	27 (27.3%)
Expanded criteria donors, *n *(%)	38 (38.4%)
Need for cardiopulmonary resuscitation, *n *(%)	21 (21.2%)
Need for antemortem intracranial surgery, *n *(%)	30 (30.3%)
Use of inotropes, *n *(%)	87 (87.9%)
Use of antidiuretic hormone, *n *(%)	60 (60.6%)
Multiorgan donors, *n *(%)	56 (56.6%)
Mean hospital days before brain death (± SD)	1.9 (± 2.1)

^a^eGFR, estimated glomerular filtration rate using the modification of diet in renal disease (MDRD) equation for glomerular filtration rate in the 96 adult donors and the Schwartz equation in three donors under 18 years of age. Expanded criteria donors are defined as all donors who were (1) over 60 years of age or (2) over 50 years of age and (3) had at least two of the following clinical characteristics: hypertension, plasma creatinine level >132 μmol/L (1.5 mg/dL) or cerebrovascular accident as the cause of death [7]. SD, standard deviation.

**Table 2:** Clinical characteristics of 176 kidney recipients and their transplantation details^a^

Clinical characteristics	Statistics
Mean age, years (± SD)	56 (56.6%)
Females, *n *(%)	66 (37.5%)
Underlying kidney disease, *n *(%)	
Polycystic disease	42 (23.8%)
Glomerulonephritis	35 (19.9%)
Diabetes mellitus	48 (27.3%)
Other	51 (30.0%)
Transplantation number, *n *(%)	
First transplantation	161 (91.5%)
Retransplantation	15 (8.5%)
Mode of pretransplantation dialysis, *n *(%)	
Hemodialysis	119 (67.6%)
Peritoneal dialysis	57 (32.4%)
Mean time of pretransplantation dialysis, days (± SD)	850 (588.8)
Mean plasma creatinine level, μmol/L (± SD)	
3 months	124 (± 51.0)
1 year	116 (± 40.6)
Mean eGFR, mL/min (± SD)	
3 months	55 (± 18.2)
1 year	58 (± 19.8)
1-year patient survival	98.9%
1-year graft survival	95.5%
Mean cold ischemia time, hours (± SD)	21.9 (± 3.70)

^a^eGFR was calculated using the MDRD equation.

### Donor S-NGAL and U-NGAL

The mean donor S-NGAL concentration was 212 ng/mL (range 27 to 720 ng/mL, SD 145.1). Donor S-NGAL concentrations correlated directly with donor plasma creatinine levels (*R*^2 ^= 0.35, *P *= 0.001) and inversely with donor eGFRs (*R*^2 ^= 0.24, *P *= 0.021).

The mean donor U-NGAL concentration was 18 ng/mL (range 0 to 177, SD 26.1). Donor U-NGAL concentrations correlated directly with donor plasma creatinine levels (*R *= 0.37, *P *< 0.0001) and inversely with donor eGFRs (*R *= 0.24, *P *= 0.01). Donor U-NGAL concentrations correlated directly with donor S-NGAL concentrations (*R *= 0.40, *P *< 0.0001).

Donors treated with ADH had significantly lower mean S-NGAL (188 ng/mL, SD 125.3) and U-NGAL (13 ng/mL, SD 14.3) levels compared to those not treated with ADH (S-NGAL: 249 ng/mL, SD 161.2, *P *= 0.002; U-NGAL: 26 ng/mL, SD 36.6, *P *= 0.045). Donor S-NGAL and U-NGAL levels did not correlate with donor age (*R *= 0.15 and *P *= NS for S-NGAL and donor age; *R *= 0.12 and *P *= NS for U-NGAL and donor age) and were not affected by gender, history of hypertension, use of vasopressors, length of hospital stay, need for cardiopulmonary resuscitation or intracranial surgery before brain death, ECD or standard criteria donor status, and multiorgan or kidney-only donation. In addition, there were no significant differences between donor S-NGAL levels in samples taken before or after brain death or before or after steroid administration (see Additional file 1).

Using the high vs. low NGAL division, we found that mean donor plasma creatinine level was significantly higher and that mean eGFR was lower in the high NGAL groups compared to the low NGAL groups (Table 3).

**Table 3:** Donor NGAL, donor kidney function and onset of graft function after transplantation^a^

Parameter	High S-NGAL (≥214 ng/mL)	Low S-NGAL (< 214 ng/mL)	*P *value	High U-NGAL (≥18 ng/mL)	Low U-NGAL (≥18 ng/mL)	*P *value
Donors, *n*	38	57		26	69	
Kidneys, *n*	69	99		52	116	
Mean donor plasma creatinine, μmol/L (± SD)	70 (22.8)	57 (± 15.1)	0.021	71 (± 21.8)	59 (± 17.8)	0.006
Mean donor eGFR, mL/min (± SD)	108 (33.9)	124 (± 34.5)	0.033	105 (± 31.2)	122 (± 35.7)	0.039
Prolonged DGF (*n *= 25)	12 (17.4%)	13 (13.1%)		12 (23.1%)	13 (11.2%)	
Short DGF (*n *= 41)	22 (31.9%)	19 (19.1%)		15 (28.8%)	26 (22.4%)	
EGF (*n *= 102)	35 (50.7%)	67 (67.8%)	NS	25 (48.1%)	77 (66.4%)	0.028
Mean recipient 1-year plasma creatinine level, μmol/L (± SD)	117 (43.8)	115 (± 37.7)	NS	114 (± 28.5)	117 (± 45.0)	NS
Mean Recipient 1-year eGFR mL/min (± SD)	57 (16.9)	60 (± 21.1)	NS	57 (± 15.9)	59 (± 21.4)	NS
1-year patient survival	98.6%	99.0%	NS	96%	100%	NS
1-year graft survival	91.4%	98.0%	0.050	90.3%	97.4%	0.048

^a^DGF, delayed graft function; EGF, early graft function; S-NGAL, serum neutrophil gelatinase-associated lipocalin; U-NGAL, urine neutrophil gelatinase-associated lipocalin.

### Donor biopsies

**Figure 1:**
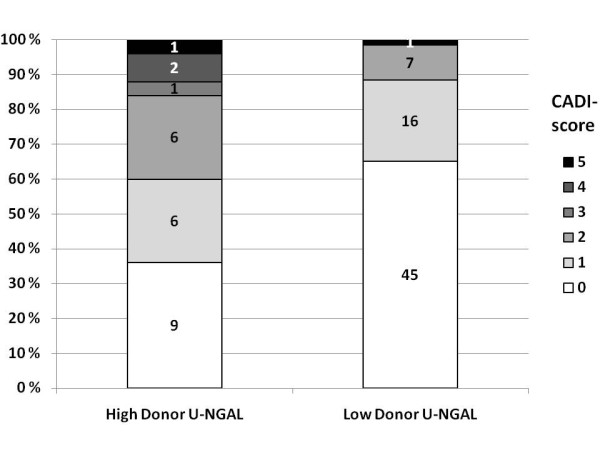
**The distribution of Chronic Allograft Damage Index (CADI) **[31]**scores of donor biopsies in the high (≥18 ng/mL) and low (< 18 ng/mL) neutrophil gelatinase-associated lipocalin (NGAL) groups**. The highest CADI score in these biopsies was 5. There were significantly more high CADI scores in the high urine NGAL (U-NGAL) group than in the low U-NGAL group (*P *= 0.010).

A representative biopsy for histological evaluation was available from 97 of 99 donors. Of the 97 biopsies, 58 (58.6%) showed normal histology. The mean CADI score of the biopsies was 0.72, ranging from 0 to 5 (Figure 1). Overall, the changes in the kidney biopsies were rare, apart from arterial changes (Table 4). Positive findings in single Banff classification components were not associated with the levels of donor plasma creatinine, eGFR, S-NGAL or U-NGAL (data not shown). However, the donors with high U-NGAL had significantly higher CADI scores than the donors with low U-NGAL (Figure 1 and Table 4).

**Table 4:** Donor kidney biopsy findings in the high and low NGAL groups^a^

	Serum NGAL			Urine NGAL		
Biopsy findings	High, ≥214 ng/mL (*n *= 38)	Low, < 214 ng/mL (*n *= 57)	*P *value	High, ≥18 ng/mL (*n *= 26)	Low, < 18 ng/mL (*n *= 69)	*P *value
Tubulitis	0 (0%)	0 (0%)	NS	0 (0%)	0 (0%)	NS
Intimal arteritis	0 (0%)	0 (0%)	NS	0 (0%)	0 (0%)	NS
Interstitial inflammation	1 (2.6%)	0 (0%)	NS	1 (3.4%)	0 (0%)	NS
Glomerulitis	0 (0%)	0 (0%)	NS	0 (0%)	0 (0%)	NS
Interstitial fibrosis	3 (7.9%)	2 (3.5%)	NS	3 (10.3%)	2 (3.0%)	NS
Tubular atrophy	3 (7.9%)	3 (5.3%)	NS	3 (10.3%)	2 (3.0%)	NS
Glomerulopathy	3 (7.9%)	3 (5.3%)	NS	3 (10.3%)	2 (3.0%)	NS
Mesangial matrix increase	1 (2.6%)	0 (0%)	NS	1 (3.4%)	0 (0%)	NS
Intimal thickening	11 (28.9%)	14 (24.6%)	NS	9 (31.0)	15 (22.7%)	NS
Arterial hyalinosis	8 (21.1%)	13 (22.8%)	NS	8 (27.5%)	15 (22.7%)	NS
CADI score, *n*						
0 or 1	30	46	NS	17	61	0.010
≥2	8	11		9	8	

^a^CADI, Chronic Allograft Damage Index [31]; NGAL, neutrophil gelatinase-associated lipocalin.

### Donor NGAL and DGF

Mean donor U-NGAL was significantly higher in cases with prolonged DGF (35 ng/mL, SD 49.4) compared to those with short DGF (15 ng/mL, SD 13.7) or EGF (15 ng/mL, SD 19.8) (*P *= 0.002). Mean donor S-NGAL did not differ significantly between the prolonged DGF (220 ng/mL, SD 141.5), short DGF (234 ng/mL, SD134.6) and EGF (206 ng/mL, SD 150.4) (*P *= NS) groups. There were no significant differences in mean donor S-NGAL and U-NGAL levels in the DGF (including both short and prolonged DGF) (S-NGAL 229 ng/mL, SD 136.4; U-NGAL 23 ng/mL, SD 33.3) and EGF (S-NGAL 206 ng/mL, SD 150.4, *P *= NS; U-NGAL 16 ng/mL, SD 19.8, *P *= 0.058) groups. High donor U-NGAL level was associated with more prolonged DGF and worse 1-year graft survival compared to low donor U-NGAL level (Table 3).

### DGF risk factors

Multivariate analysis was performed to assess the factors predicting DGF and prolonged DGF. We included in the multivariate analysis the factors differing significantly between the DGF and EGF groups (donor age, ECDs vs. standard criteria donors, cold ischemia time and recipient pretransplantation mode of and time on dialysis) in addition to donor S-NGAL, U-NGAL, eGFR and plasma creatinine levels.

**Figure 2:**
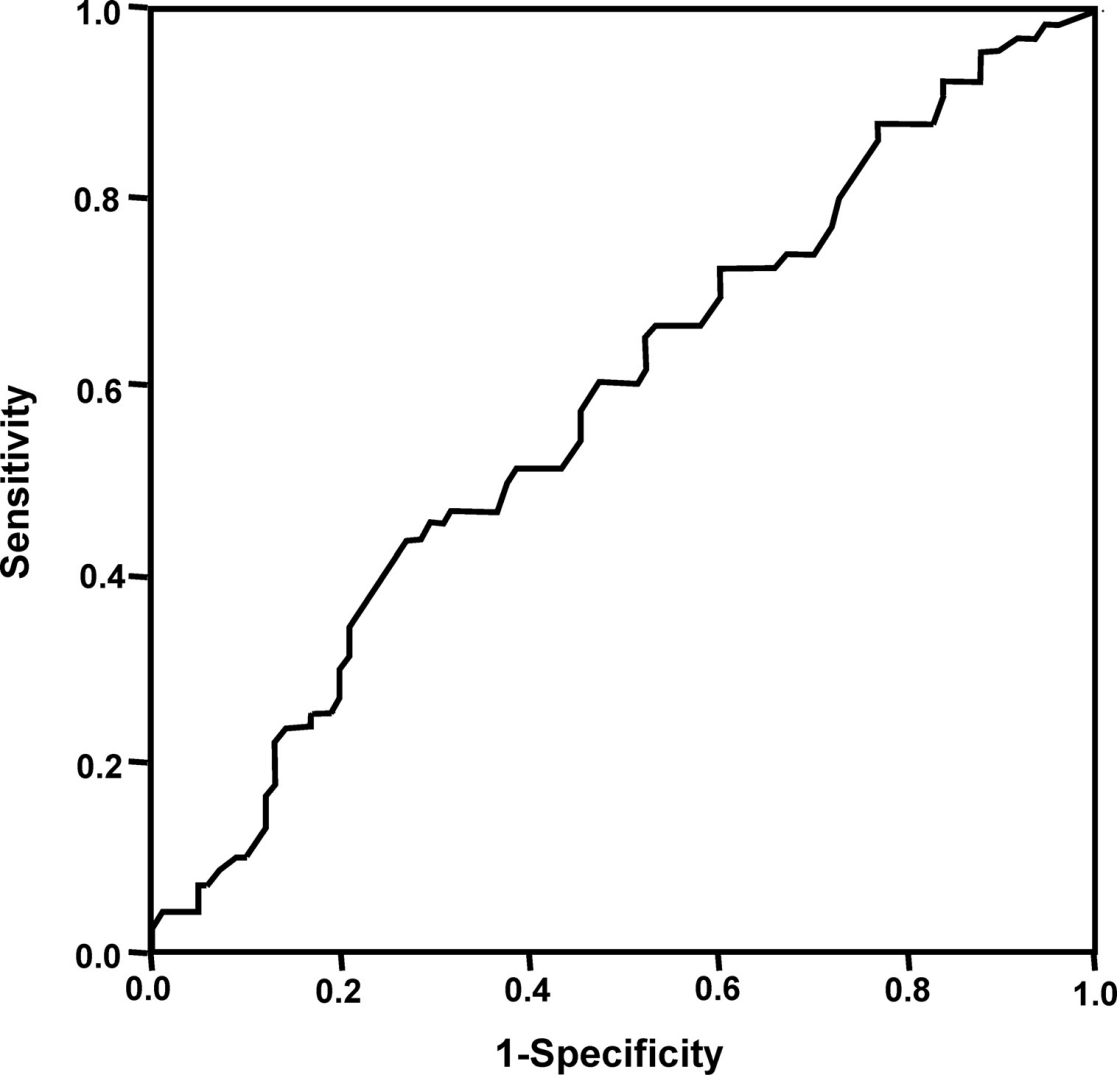
**Receiver operating characteristic curve (ROC) analysis of donor U-NGAL in predicting delayed graft function after kidney transplantation**.

**Figure 3:**
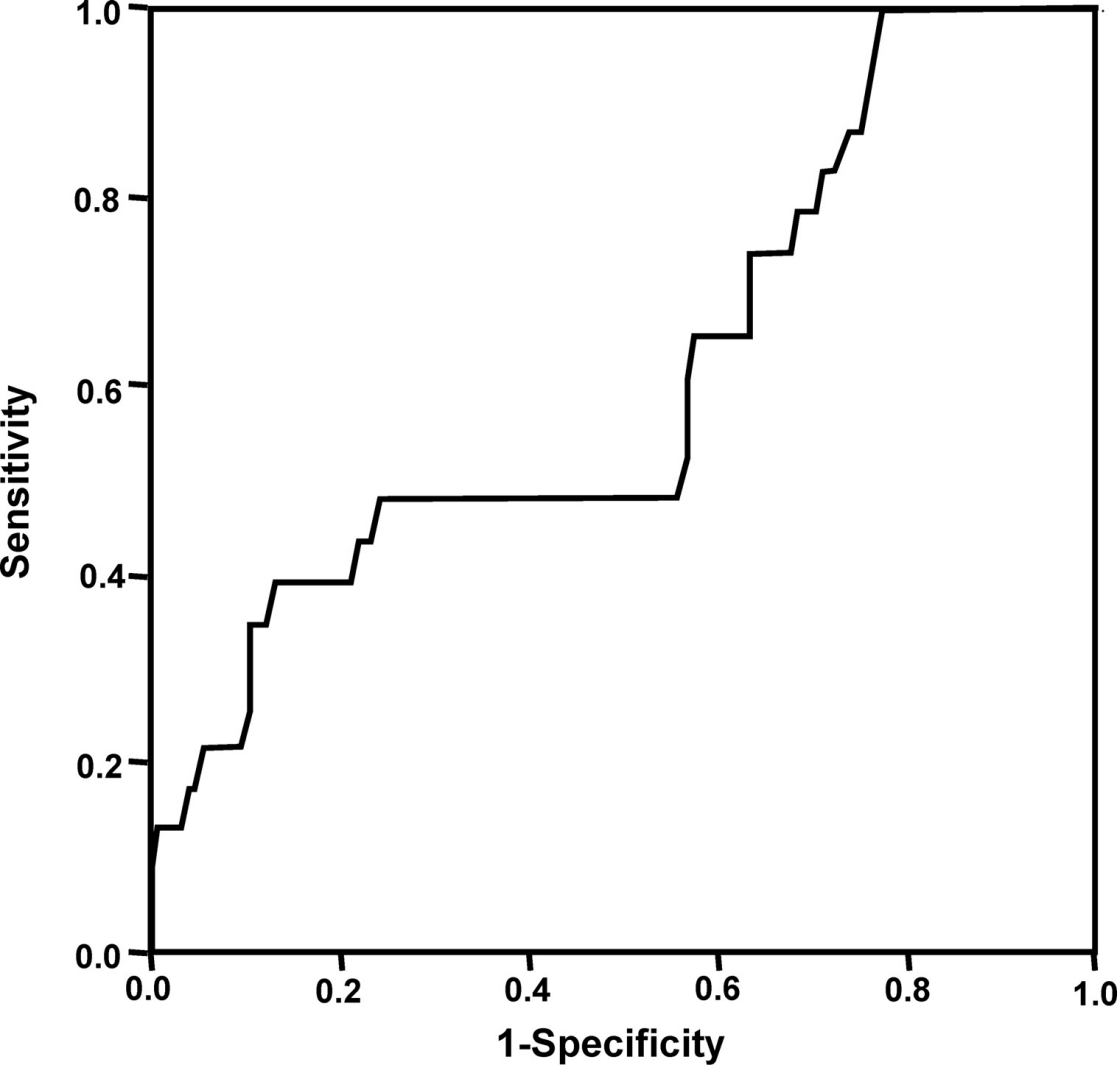
**ROC analysis of donor U-NGAL in predicting prolonged, delayed graft function (longer than 14 days) after transplantation**.

None of the included factors appeared to be a significant risk factor for DGF *per se*. Donor U-NGAL, ECD status and eGFR emerged as independent risk factors for prolonged DGF (Table 5). ROC analysis for donor U-NGAL in predicting DGF (Figure 2) resulted in an area under the curve (AUC) of 0.595 (95% confidence interval (95% CI) 0.506 to 0.749). ROC analysis performed to predict prolonged DGF (Figure 3) resulted in an AUC of 0.616 (95% CI 0.493 to 0.739). Table 6 shows the sensitivities, specificities and positive and negative predictive values at the lowest quartile (4 ng/mL), the median (9 ng/mL), the mean (18 ng/mL) and the highest quartile (20 ng/mL).

**Table 5:** Multivariate analysis of prolonged DGF predictors^a^

Clinical characteristics	*P *value
Donor age, years	0.523
Donor urine NGAL, ng/mL	0.001
Donor serum NGAL, ng/mL	0.096
Donor plasma creatinine, μmol/L	0.152
Donor eGFR, mL/min	0.016
Expanded criteria donors	0.038
Cold ischemia time, hours	0.066
Mode of dialysis, hemodialysis or peritoneal dialysis	0.321
Time on dialysis before transplantation, days	0.460

^a^Expanded criteria donors were defined according to the criteria outlined by Port *et al. *[7].

**Table 6:** Sensitivity and specificity at different cutoff values in U-NGAL receiver operating characteristic curve analysis predicting DGF and prolonged DGF^a^

U-NGAL cutoff level, ng/mL	DGF				Prolonged DGF			
	Sensitivity	Specificity	PPV	NPV	Sensitivity	Specificity	PPV	NPV
4	77.3%	28.4%	0.42	0.65	82.6%	27.6%	0.16	0.91
9	60.6%	54.9%	0.47	0.68	47.8%	49.7%	0.14	0.85
18	37.9%	77.5%	0.53	0.65	47.8%	74.5%	0.24	0.90
20	34.8%	79.4%	0.53	0.65	43.5%	76.6%	0.24	0.89

^a^NPV, negative predictive value; PPV, positive predictive value. The selected cutoff values are the lowest quartile (4 ng/mL), the median (9 ng/mL), the mean (18 ng/mL) and the highest quartile (20 ng/mL).

A pair kidney analysis was possible in 77 donors (154 kidneys). In 28 of 77 cases both donated kidneys had EGF, in 13 of 77 cases both kidneys had DGF and in 36 of 77 cases one of the kidneys had DGF and the other had EGF. If one kidney had DGF, the other was not at increased risk for DGF (*P *= NS).

## Discussion

In kidney transplantation, the donor issues have become more important because, owing to a shortage of organs, many donor kidneys which earlier would have been discarded are now accepted for transplantation. DGF complicates a significant amount of kidney transplantations from deceased donors, and the rate of DGF is expected to increase as more ECD kidneys are used [3,4]. It is generally known that plasma creatinine is a poor marker of AKI, especially when donor is in an unstable state, and thus a test revealing the quality of donor kidneys already at the time of donor evaluation would be extremely welcome.

The gold standard for GFR determination is measurement of insulin clearance. For practical reasons, it is impossible to perform this test in a deceased donor. Estimated GFR and plasma creatinine level are the only readily available tools to assess donor kidney function. In clinical practice, GFR is estimated by using different equations, among which the MDRD equation [28] is the most widely used. It is common knowledge that to obtain reliable results, GFR should be calculated in a stable situation. As the donors are not in a steady state, the eGFRs and plasma creatinine concentrations must be regarded only as approximate measures of kidney function.

NGAL is a promising biomarker of AKI, and it has been demonstrated to be useful in many clinical situations [11-22]. In kidney transplant recipients, s-NGAL and U-NGAL concentrations have been shown to predict DGF [23-27], but the literature on NGAL in kidney transplantation is limited. As far as we know, there are no previously published data on NGAL in deceased organ donors. The NGAL levels of our donors corresponded well to the levels reported in several patient groups treated in ICUs [17-20,34].

NGAL is an acute phase protein [35], and it is abundant in human neutrophils and macrophages [36]. NGAL is induced by a range of cytokines [37-39]. Brain death causes a large cytokine storm and inflammatory response in the donor, and brain death, together with other factors associated with donor death, might thus explain the high levels of S-NGAL in our donors with apparently healthy kidneys. None of our donors had clinically verified AKI before death, and all appeared to have good kidney function according to their plasma creatinine levels and eGFRs. Furthermore, the findings in donor biopsies taken before initiating *in situ *perfusion were meager.

The S-NGAL and U-NGAL concentrations were analyzed using different methods. At the time the laboratory analyses were performed, only the ELISA and ARCHITECT NGAL methods were commercially available. The ARCHITECT method is only available for U-NGAL analysis. In clinical practice, the NGAL detection method has to be simple, easy to use, quick and robust; hence the ELISA method is not optimal. Since then, a point-of-care method of S-NGAL detection has become available. Because of the use of different measurement methods, the U-NGAL and S-NGAL levels reported in this paper are not directly comparable.

The U-NGAL levels reported in this study were in general low, corresponding to the levels of healthy individuals [40]. It has previously been suggested that U-NGAL is likely to originate from the kidney [41]. Thus, high U-NGAL concentration in the donor is suggestive of local damage in the kidney and seems to be more specific to AKI compared to S-NGAL, which also may originate from other organs such as the lungs, bone marrow and gastrointestinal tract [36]. It is thus likely that the high S-NGAL levels detected in the donors did not originate from the kidneys only, but from other sites as well. Circulating NGAL is filtered through the glomerulus and reabsorbed in the proximal tubule, where it is degraded. NGAL detected in the urine is believed to derive mainly from tubular epithelial cells, where it is synthesized *de novo *as a response to AKI [42,43]. However, some of the NGAL detected in the urine can also be derived from other organs. So far, it has not been possible to trace the origin of measured NGAL in the urine in a clinical situation.

Diabetes insipidus is sometimes seen as a consequence of brain death. Interestingly, we noticed significantly lower U-NGAL levels in donors who had needed ADH treatment because of massive postmortem polyuria. ADH regulates urine volume and concentration and may improve renal perfusion pressure. It does not result in increased GFR. We can speculate that ADH treatment causes a decrease in *de novo *tubular NGAL synthesis, but the mechanism behind that remains unclear. We can also speculate that increased renal perfusion pressure may result in better kidney function and less damage and hence lower NGAL levels. On the other hand, in addition to ADH treatment, diuretic use may affect U-NGAL levels.

As expected, the pathological findings in the donor biopsies were few, and thus it was not possible to demonstrate a significant correlation between single pathological changes in the biopsies and NGAL levels. However, there were significantly higher CADI scores in the high U-NGAL group compared to the low U-NGAL group. This may indicate that kidneys with preexisting chronic changes as shown by the CADI score are susceptible to injury during the brain death process. This difference was not seen in the high vs. low S-NGAL groups, again supporting the suggestion that U-NGAL might be a better and more specific marker for AKI than S-NGAL.

Mean donor S-NGAL and U-NGAL levels were rather similar between the DGF and EGF groups. However, high U-NGAL concentration in the donor was associated with more DGF and, in addition, with a worse outcome after transplantation, despite the fact that U-NGAL levels in the majority of the donors remained low. The etiology of DGF is multifactorial, and it is thus possible that the effect of NGAL is concealed by other factors associated with DGF, such as cold ischemia time, donor age and ECD status. This also explains the result of our pair analysis.

Prolonged DGF is a clinically relevant risk factor for long-term kidney graft survival [1,2], as shown also in this study. Donor U-NGAL was significantly higher in transplantations with prolonged DGF compared to those with early function or only short DGF, and donor U-NGAL was also an independent risk factor for prolonged DGF in the multivariate analysis. However, U-NGAL failed to show predictive power in the ROC analysis. Donor U-NGAL level seems to reflect the quality of the donor kidney; kidneys from donors with higher U-NGAL levels were more susceptible to ischemia-reperfusion injuries and had less reserve capacity to tolerate stress.

Our study has certain limitations. We did not examine other relevant biomarkers in parallel, which would have been valuable in the evaluation of the NGAL results. The possible confounding effects of donor treatment on NGAL concentration and NGAL analyses are not known and thus could not be eliminated. Our study also has strengths. It is the first prospective study to examine S-NGAL and U-NGAL levels in deceased kidney donors. This study comprised consecutive deceased donors and represents well our general deceased donor population.

## Conclusions

This is the first report on S-NGAL and U-NGAL levels in deceased kidney donors. Kidneys from donors with high U-NGAL values had significantly more prolonged DGF and more histological findings (higher CADI scores) in donor biopsies, but the predictive power of U-NGAL with regard to the onset of graft function was weak. Donor S-NGAL levels did not have predictive power with regard to the onset of graft function after transplantation. Donor U-NGAL level, but not S-NGAL level, is useful when evaluating a potential deceased organ donor.

## Key messages

• Deceased donor U-NGAL concentration is useful when evaluating the kidneys of potential organ donor candidates in the ICU.

• The mean S-NGAL concentration (determined by ELISA) in deceased donors was 212 ng/mL, corresponding to the previously reported levels in critically ill patients.

• The mean U-NGAL concentration (determined by the ARCHITECT method) was 18 ng/mL, which was well within the range of healthy controls.

• Deceased donor U-NGAL concentration is more specific than S-NGAL concentration to kidney injury.

• High deceased donor U-NGAL concentration was associated with prolonged oliguria after kidney transplantation and chronic histological changes in donor kidney biopsies, but it was a poor predictor of DGF in individual cases.

## Abbreviations

ADH: antidiuretic hormone; AKI: acute kidney injury; CADI: Chronic Allograft Damage Index; DGF: delayed graft function; ECD: expanded criteria donor; eGFR: estimated glomerular filtration rate; EGF: early graft function; MDRD: modification of diet in renal disease equation for glomerular filtration rate; NGAL: neutrophil gelatinase-associated lipocalin; S-NGAL: serum neutrophil gelatinase-associated lipocalin; SD: standard deviation; U-NGAL: urine neutrophil gelatinase-associated lipocalin.

## Competing interests

One author of this manuscript has conflicts of interest to disclose. MEH was sponsored by Abbott Diagnostics to the American Transplant Congress in 2010, where an oral presentation of this study was presented. She also received an honorarium for presenting the recipient U-NGAL data [22] in a meeting organized by Abbott Diagnostics. The other authors of this manuscript have no conflicts of interest to disclose.

## Authors' contributions

MEH collected data, carried out S-NGAL analyses, analyzed data and wrote the paper. LEK designed study, analyzed data and wrote the paper. KAI carried out U-NGAL analyses. MLTL and JM designed study. KTS designed study and wrote the paper. All authors read and approved the final manuscript.

## Supplementary Material

Additional file 1 **Donor serum and urine neutrophil gelatinase-associated lipocalin (U-NGAL) and donor parameters**. The additional data file shows the association between donor parameters and serum NGAL and U-NGAL concentrations. Click here for file
