# Supplementary material for: Deceased donor neutrophil gelatinase-associated lipocalin and delayed graft function after kidney transplantation: a prospective study
Source: Crit Care. 2011 May 5;15(3):R121. doi: 10.1186/cc10220 (PMC3218974; doi:10.1186/cc10220)
Supplement: Additional file 1 — Donor serum and urine neutrophil gelatinase-associated lipocalin (U-NGAL) and donor parameters. The additional data file shows the association between donor parameters and serum NGAL and U-NGAL concentrations. [file cc10220-S1.DOC]

Additional files provided with the submission

Donor serum and urine NGAL (Neutrophil gelatinase-associated lipocalin) and donor parameters

|  | **Serum NGAL ng/mL (SD)** | ***p-value*** | **Urine NGAL ng/mL (SD)** | ***p-value*** |
| --- | --- | --- | --- | --- |
| **Gender**  Female (43.4%)  Male (56.6%) | 196 (148.4)  225 (142.9) | *NS* | 18 (35.5)  19 (17.8) | *NS* |
| **History of hypertension**  Yes (27.3%)  No (72.7%) | 176 (95.4)  226 (157.5) | *NS* | 13 (15.3)  20 (29.1) | *NS* |
| **Need for ante mortem surgery**  Yes (30.3%)  No (69.7%) | 197 (142.8)  219 (145.9) | *NS* | 14 (17.6)  20 (29.0) | *NS* |
| **Cardiopulmonary resuscitation**  Yes (21.2%)  No (78.8%) | 240 (130.5)  204 (148.2) | *NS* | 15 (12.8)  19 (28.5) | *NS* |
| **Use of vasopressors**  Yes (87.9%)  No (12.1%) | 214 (148.8)  180 (105.6) | *NS* | 17 (19.5)  29 (56.5) | *NS* |
| **Use of ADH**  Yes (60.6%)  No (39.4%) | 188 (125.3)  249 (161.2) | *p=0.002* | 13 (14.3)  26 (36.6) | *p=0.045* |
| **Cause of death**  Cerebrovascular accident (74.7%)  Traumatic brain injury (25.3%) | 230 (156.1)  206 (141.1) | *NS* | 17 (27.4)  20 (21.8) | *NS* |
| **Multiorgan donation**  Yes (56.6%)  No (43.4%) | 216 (144.6)  207 (146.9) | *NS* | 18 (30.6)  17 (18.5) | *NS* |
| **Expanded criteria donor**  Yes (38.4%)  No (61.6%) | 208 (123.8)  206 (157.5) | *NS* | 16 (16.7)  19 (30.7) | *NS* |
| **Sampling time relative to brain death**  Before the diagnosis (36.4%)  After the diagnosis (63.6%) | 225 (169.4)  205 (129.3) | *NS* | Not applicable |  |
| **Sampling time relative to steroid admission**  Before (77.8%)  After (22.2%) | 198 (142.8)  214 (150.2) | *NS* | Not applicable |  |

*NGAL=neutrophil gelatinase-associated lipocalin, ADH=anti diuretic hormone, SD=standard deviation. Expanded criteria donors were defined according to Port et al. (7).*
